# 340B Contract pharmacy growth by pharmacy ownership: 2009–2022

**DOI:** 10.1093/haschl/qxad075

**Published:** 2023-12-09

**Authors:** Claire McGlave, John P Bruno, Elizabeth Watts, Sayeh Nikpay

**Affiliations:** Division of Health Policy and Management, University of Minnesota, Minneapolis, MN 55455, United States; Department of Economics, University of Minnesota, Minneapolis, MN 55455, United States; Division of Health Policy and Management, University of Minnesota, Minneapolis, MN 55455, United States; Division of Health Policy and Management, University of Minnesota, Minneapolis, MN 55455, United States

**Keywords:** 340B, pharmacy ownership, access to care

## Abstract

The 340B program grants eligible health care providers (“covered entities”) access to discounted prices for outpatient prescription drugs. Covered entities frequently rely on retail pharmacies (“contract pharmacies”) to dispense discounted drugs. This analysis describes contract pharmacy participation by ownership: the top 4 chains, grocery chains, small chains, and institutional independent pharmacies. We found that 71% of pharmacies in the top 4 chains were contract pharmacies. Forty one percentage of institutional pharmacies, 38% of grocery store pharmacies, and 22% of independent pharmacies participated in 340B in 2022. The median number of contracts per pharmacy was 2 among the top 4 chains and grocery store pharmacies vs 1 for all other pharmacy types. The median farthest distance in miles from contracting covered entities was largest for the top 4 chains (19 miles) and small chains (18 miles) and smallest for independent and institutional pharmacies (10 miles). The top 4 chains held the highest proportion of contracts with core safety-net providers (75% vs 61% of institutional pharmacies).

## Introduction

Uninsured and underinsured patients face difficulties affording health care, frequently turning to the health care safety net to meet their medical needs. The health care safety net is an informal network of hospitals and clinics that provide care regardless of ability to pay.^1^ Since 1992, the 340B program has aimed to support the health care safety net through significant discounts on prescription drugs. The program both lowers operating costs for organizations that provide free or sliding-scale drugs to patients, and allows providers to bill insurers usual and customary rates for discounted drugs, generating 340B revenue to “stretch scarce federal resources” and thereby fund access to safety-net care.^2^ As a result, covered entities benefit most from the program when drugs are prescribed to patients covered by Medicare and commercial insurance.

340B drugs represent a large portion of the US prescription drug market. In 2021, hospitals paid almost $44 billion for 340B drugs, which, if purchased at list prices, would have totaled $94 billion.^3^ In 2020, 340B purchases represented more than 7% of spending in the US prescription drug market.^4^ Discounted drugs can be dispensed through either clinics and on-site pharmacies, or since 2010, in an unlimited number of off-site retail and specialty pharmacies. In return for dispensing discounted drugs on behalf of the 340B provider, these pharmacies, called “contract pharmacies,” earn a fee for each prescription dispensed or a portion of the 340B revenue generated by drug sales to 340B patients.^5^

340B contract pharmacy arrangements have grown significantly over the past 13 years. In 2010, less than 1% of pharmacies participated in contract arrangements, expanding to over 40% by 2022.^6,7^ Over time, retail pharmacies contracting with 340B participants were more likely to have multiple contracts with health care facilities that were less safety-net engaged and located farther away.^7^ Most communities in the United States now contain at least 1 contract pharmacy, and several studies document that 340B participants, especially hospitals, establish contracts in areas with fewer uninsured and more wealthy patients.^6,8,9^ These results are consistent with participants seeking to maximize 340B revenue and are similar to growth patterns for 340B clinics.^10^

Concern over growing contract pharmacy arrangements has centered around access to care for safety-net patients, the potential for violation of program rules, and diversion of 340B revenue for purposes other than funding safety-net care. Access concerns are motivated by a recent report by the Government Accountability Office, which found that approximately half of 340B contract pharmacy arrangements did not extend discounts to uninsured patients.^5^ As a result, 340B providers might generate 340B revenue off of safety-net–reliant patients, imposing unnecessary financial burden on the very population the program seeks to assist. Violation of program rules stems from the inability of pharmacies to accurately determine whether customers are eligible patients of a 340B provider or covered by Medicaid, for which discounts are already granted through the Medicaid Drug Rebate Program.^11^ Finally, diversion of 340B savings away from safety-net care is motivated by a recent New York Attorneys General lawsuit against a large pharmacy chain operating a large share of 340B contract pharmacies, alleging it charged exorbitant fees, diminishing the amount of 340B revenue available to enhance safety-net care.^12^ Although the suit focused on 1 chain, reporting on 340B contract pharmacies suggests that more than three-quarters of 340B contracts are dominated by a handful of large pharmacy chains. Market analyses suggest that the 3 largest chains participating in 340B generated $2.2 billion in revenue from 340B in 2021.^13,14^

At the same time as contract pharmacy arrangements appear to be growing among large chains, rural and independent pharmacies have been closing.^15,16^ These pharmacies are more likely to serve safety-net–reliant patients than other retail pharmacies^17^; however, the extent to which independent pharmacies participate in the program is not known.

In this research brief we describe the role of chain and independent retail pharmacies in the 340B program by presenting trends in measures of 340B contract pharmacy participation over time and across categories of ownership.

## Data and methods

We linked the 2023 Health Resources and Services Administration’s (HRSA’s) Office of Pharmacy Affairs (OPA) database to the 2009–2022 National Council for Prescription Drug Program (NCPDP) data. The OPA database contains information on all pharmacy contracts between 340B program participants and pharmacies from the program’s inception to the present. We used the name and address of the pharmacy as recorded by each 340B participant to link pharmacies in the database to the National Plan and Provider Enumeration System—the Centers for Medicare and Medicaid Services’ unique national provider identifier (NPI)—using text and geographic matching algorithms.^7^ We then linked each pharmacy in the OPA database to retail pharmacies in the NCPDP data using the NPI. Retail pharmacy status was defined as having a primary provider type code in the NCPDP indicating retail and a secondary provider type that was not specialty or mail order. The resultant dataset yielded 182 021 pharmacy-year observations over the 2009–2022 study period.

We constructed 4 measures of participation in 340B contracts using the OPA database following studies in the literature.^6,7^ These included an indicator for whether the pharmacy participated in any contract pharmacy arrangement (participation), a count variable equal to the number of contracts held by the pharmacy in each year (depth), the maximum geographic distance between each pharmacy and the 340B participants it contracts with (spread), and finally, an indicator for whether contract pharmacies have no contracts with core safety-net providers (safety-net composition). Safety-net providers are classified as federal grantees or hospitals that meet the Medicaid and CHIP (Children’s Health Insurance Program) Payment and Access Committee’s (MACPAC) definition of essential community hospitals. Although multiple definitions of safety-net status exist for US hospitals, we chose to rely on MACPAC’s definition as it was possible to construct it from publicly available data and it has been used for almost a decade in reports to Congress on safety-net hospitals. Other definitions could define more or fewer hospitals as safety-net providers.

We also constructed a pharmacy ownership variable with 8 mutually exclusive categories based on NCPDP data and informed by existing studies in the literature.^9^ Specifically, we used dispenser class codes and parent company names to assign each pharmacy to the top 4 chains by volume (Walgreens, CVS, Walmart, RiteAid), grocery store pharmacies (such as Kroger), institutional pharmacies (defined as based in a health care provider), and independent pharmacies (defined as pharmacies with 3 or fewer locations). Pharmacies not fitting into these categories were considered to be “other small chains.” Upon review of the data we found that pharmacies falling into this last, residual category were most often small chains and franchised pharmacies (such as Medicine Shoppe). For pharmacies that fit into multiple categories, we assigned the pharmacy to the larger category. For example, pharmacies that were indicated as independent in NCPDP but also had a parent company name indicating a grocery store were assigned to the grocery store category.

We present descriptive statistics comparing each of the four 340B contract pharmacy participation measures against pharmacy ownership. We present medians and interquartile ranges for measures exhibiting skew and means and standard deviations for other measures. We performed analysis of variance tests to assess whether participation rates differed across ownership categories and assessed statistical significance at *P* = .05. The analysis was performed using Stata 18 (StataCorp).

## Results

**Figure 1.**
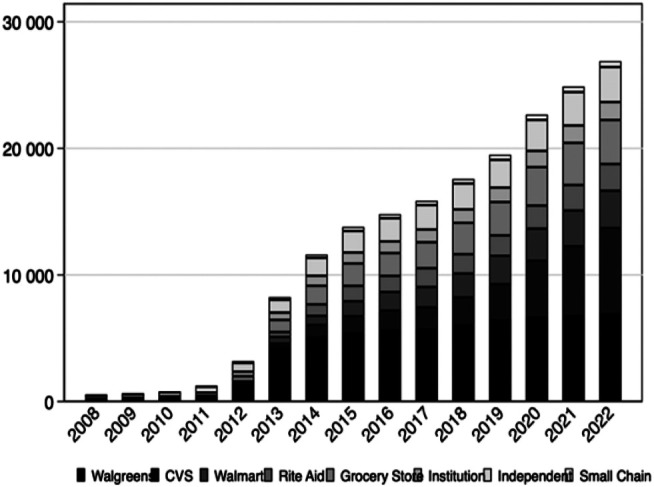
Trends in 340B participation by chain status (2009–2022). Bars represent the number of retail pharmacies with 340B contracts per chain in each year. *n* = 388 324 contract-years, representing a total of 33 951 unique pharmacy locations.

In 2009, there were a total of 601 retail pharmacies participating in the program—9% of these were owned by Walgreens. By 2022, the number of pharmacies participating was 26 885, and the majority were still pharmacies owned by Walgreens (26%), closely followed by CVS (25%), Walmart (11%), and Rite Aid (8%) (Figure 1). Grocery store chains accounted for 13%, institutional pharmacies accounted for 5%, independent pharmacies 10%, and small chains accounted for 2% of pharmacies participating in 340B in 2022.

Among the top 4 chains, (Walmart, CVS, Rite Aid, and Walgreens), 71% of locations held 340B contracts in 2022, compared to 22% of independently owned pharmacy locations and 38% of grocery store pharmacy locations (Table 1). However, among the top 4 chains, 78% of Walgreens locations held contracts with covered entities in 2022, followed by 68% of CVS locations, 57% of Walmart locations, and 84% of Rite Aid locations. This large rate of Rite Aid participation may be inflated by the relatively small number of total locations (less than half that of other top 4 pharmacies).

**Table 1.** Mean depth, safety-net composition, and spread by chain status (2022).

	Total US pharmacies	No. with 1+ contracts	Proportion of locations with 1+ contracts	Proportion of all contracts	Depth, median (IQR)	Geographic spread, median (IQR)	Proportion of contracts with safety-net providers, mean (SD)
Top 4 chains	26 490	18 761	71%	78%	2 (1–4)	19 (8–39)	75% (34%)
Grocery stores	9053	3481	38%	9%	1 (1–2)	12 (5–27)	63% (42%)
Small chains	1102	453	19%	1%	2 (1–3)	18 (4–34)	49% (44%)
Institution	6504	1418	41%	3%	1 (1–2)	10 (1–27)	61% (45%)
Independent	14 767	2772	22%	8%	1 (1–2)	10 (1–26)	69% (44%)

The table shows the count of all pharmacies in the United States, regardless of 340B participation, the number of pharmacies with at least 1 contract, the proportion of each type of pharmacy’s sites with at least 1 contract, and the proportion of total contracts between covered entities and each type of pharmacy. The table also displays the median and interquartile range (IQR) of the number of contract depth (number of contracts per site) and geographical spread (distance from the farthest contract pharmacy in miles). The final column presents the mean and standard deviation (SD) of safety-net composition (the share of each pharmacy types’ contracts with core safety-net providers). The top 4 chains include the top 4 retail chains by market share (Walgreens, CVS, Walmart, and Rite Aid), grocery store chains, small chains, institutional pharmacies, and independent pharmacies. *n* = 57 916 for all pharmacies in the United States, *n* = 26 980 for pharmacies participating in 340B.

Average depth, or the number of contracts per pharmacy, differed across chains (*P* < .001) but did not appear to be related to chain size. Median depth was highest for top 4 pharmacies and small chains (2 contracts per pharmacy); and remained at 1 contract for all other types of pharmacies. Pharmacies differed in the proportion of contracts with core safety-net providers (*P* < .001): among the top 4 chains, an average of three-quarters of contracts were with core safety-net providers, compared to 63% of grocery store pharmacy contracts, half of institutional contracts, and 61% of independent pharmacy contracts. It is important to note, however, that the high proportion of contracts with core safety-net providers among the top 4 chains is primarily motivated by Walgreens, which has a high concentration of contracts with core safety-net providers (mean = 78%). Geographic spread also differed across chain size (*P* < .001) and was highest among the top 4 pharmacies (median distance = 19 miles), followed closely by the small chain pharmacies (median = 18 miles). The median maximum distance between a pharmacy and covered entity for independent pharmacies was 10 miles, compared to 12 miles for grocery stores.

Pharmacy participation in 340B varies widely by chain but is dominated by large chains. Among these chains, Walgreens and CVS have a disproportionately large role in the program relative to their share of total retail pharmacies: Walgreens and CVS hold 33% and 30% of 340B pharmacy contracts, respectively, yet represented only 15% and 17% of all retail pharmacies in 2022. The majority of Walgreens retail pharmacies participate in 340B (78%), in addition to over half of CVS locations (68%). A large share of Rite Aid locations participate in 340B (57%), but they have far fewer pharmacies overall, so their number of locations participating is still much smaller than any of the other top 4 pharmacies. Among the 4 largest pharmacy chains (Walgreens, CVS, Walmart, and Rite Aid), Walgreens held the most contracts in 2022 (25 303) compared with CVS, which followed with 23 021. Walgreens had the largest proportion of contracts with core safety-net providers (78%) compared to 74%, 71%, and 68% from CVS, Walmart, and Rite Aid, respectively. Finally, CVS had the largest median geographic spread (21 miles). That means that the median distance between a CVS pharmacy and the health care facility with which they were contracting was 21 miles, compared to a median of 10 miles for independent pharmacies and 12 miles for grocery store pharmacies.

## Discussion

We show that the increase in the proportion of retail pharmacies that have entered into a contract with a 340B participant is largely driven by the 4 largest chains: Walgreens, CVS, Walmart, and Rite Aid. Of these chains, Walgreens pharmacies were early participants and comprised a significant share of contracting pharmacies since 2010. These 4 firms also hold a disproportionately large share of the contracts relative to their share of physical locations. In contrast, independent pharmacy participation did not increase over time and, in 2022, these pharmacies had a disproportionately small share of contracts relative to their share of US retail pharmacies. Given the financial benefits to pharmacies participating in this program, lower participation among pharmacies that are at higher risk of closure is concerning.^18^ Independent, rural pharmacies are also more likely to serve in a safety-net role, and are therefore more closely aligned with the 340B program’s goals.^17^

Despite these differences in the likelihood of participating, once pharmacies establish at least 1 contract, they do not differ substantially across the number of current contracts. The median number of contracts per pharmacy was approximately twice as high for the top 4 pharmacy chains and grocery stores as the number for other pharmacy ownership types. However, the largest retail pharmacies tended to have contracts that were located farther away than independent pharmacies. Interestingly, the top 4 chains and independent pharmacies were similar in the proportion of contracts with core safety-net providers. High safety-net composition among the top 4 chains may reflect the fact that these pharmacies are more likely to be located in urban areas where both safety-net and non–safety-net providers operate.

Our study had a number of limitations. First, our results are limited by a lack of access to prescription volumes. Therefore, we cannot infer the number of discounted prescriptions distributed through each pharmacy and the economic value of the 340B program. Second, the study is based on retail pharmacies and may not be generalizable to specialty pharmacies, which comprise a growing share of prescriptions nationwide.^19^ Third, we relied on NCPDP data to identify pharmacy ownership type, which may be reported with error. Fourth, our definition of core safety-net providers relies on 1 of several possible definitions. We selected the Medicaid and CHIP Payment Access Commission’s definition given its frequent use by policy advisory bodies and data accessibility.^20-22^

## Conclusion

Pharmacy chains have benefited from the expansion of 340B contract pharmacy arrangements, raising concerns about the feasibility of participation from independent pharmacies. The benefits of 340B participation for pharmacies include fees, which the Government Accountability Office has found typically include a dispensing fee or sometimes a portion of 340B revenue.^5^ In the case of the large chain pharmacies, their significant market clout could result in higher negotiated fees for serving as a contract pharmacy, all else being equal.

As several studies suggest that 340B revenue may not further access to care for uninsured and underinsured populations,^23,24^ drug manufacturers have challenged the legality of HRSA’s 2010 expansion of contract pharmacies. As of 2023, over 20 manufacturers have refused to grant 340B discounts for drugs dispensed through contract pharmacies unless special conditions are met.^25^ Therefore, whether the growth in contracts will yield growth in 340B savings to core safety-net providers is an open question for researchers.

## Supplementary Material

qxad075_Supplementary_Data
